# Thermal Degradation of Carotenoids from Jambu Leaves (*Acmella oleracea*) during Convective Drying

**DOI:** 10.3390/foods12071452

**Published:** 2023-03-29

**Authors:** Jardilene da Silva Moura, Railson Pontes e Sousa, Luiza Helena da Silva Martins, Carlos Emmerson Ferreira da Costa, Renan Campos Chisté, Alessandra Santos Lopes

**Affiliations:** 1 Programa de Pós-Graduação em Ciência e Tecnologia de Alimentos (PPGCTA), Instituto de Tecnologia (ITEC), Universidade Federal do Pará (UFPA), Belém 66075-110, PA, Brazil; 2 Faculdade de Biotecnologia, Universidade Federal do Pará (UFPA), Belém 66075-110, PA, Brazil; 3 Instituto de Saúde e Produção Animal (ISPA), Universidade Federal Rural da Amazônia (UFRA), Belém 66077-830, PA, Brazil; 4 Laboratório de Óleos da Amazônia (LOA), Instituto de Ciências Exatas e Naturais (ICEN), Universidade Federal do Pará (UFPA), Belém 66075-110, PA, Brazil

**Keywords:** amazonian vegetables, β-carotene, kinetics, lutein

## Abstract

Jambu (*Acmella oleracea*) is a vegetable used in human food. Drying is an alternative to increase the shelf life of the product. High temperatures can induce the degradation of carotenoids and reduce the health benefits of these compounds. This study investigated the effect of the Jambu leaves’ drying temperature on the carotenoid composition. It was performed previously by screening 16 plants from different localities based on the total carotenoid content. The process of drying by convection was carried out at temperatures of 35, 40, 50, and 60 °C in an air circulation oven, at an air velocity of 1.4 m/s^−1^ and a processing time of ~20 h. The drying data were fitted to six mathematical models and the quantification of the carotenoid retention was determined by HPLC-DAD. The study demonstrates that the carotenoid content among the samples collected from the 16 producers varied by 72% (lower—175 ± 16 μg/g, higher—618 ± 46 μg/g). Among the models, the Page model was found to be the most suitable model to explain the variation of the experimental data. The drying process at 40 °C reduces the Jambu leaves’ carotenoid content significantly (*p* < 0.05) (All-*trans*-β-carotene—86 ± 2 μg/g, All-*trans*-lutein—141 ± 0.2 μg/g) but does not alter the carotenoid profile. The occurrence of similar reduction behavior was observed for the different carotenoids at all the temperatures studied. The drying process at 35 °C was the condition that ensured the highest retention of carotenoids, and also a product classified as a very high source of carotenoids (total carotenoids—748 ± 27 μg/g, vitamin A—17 ± 1 μg RAE/g). Thus, this study concludes that a temperature of 35 °C for 14 h (air velocity—1.4 m/s^−1^) is the best drying condition for Jambu leaves using a low-cost dryer and as a possibility for the preservation and marketing of this Amazonian raw material.

## 1. Introduction

*Acmella oleracea* (L.) R.K. Jansen, popularly known as Jambu, is a species from the Amazon region with strong commercial appeal, mainly due to spilanthol in the plant’s inflorescences. This compound gives the vegetable a flavour with functional properties and astringent characteristics. The leaves have about 89 to 93% water, making it an extremely perishable product [1,2].

For products with high water content, conservation by convective drying is still widely used due to its low cost and operational ease. Post-harvest drying is essential to preserve quality for long periods and increase the commercial expansion of the product [3]. However, it is a process that involves significant losses of important bioactive compounds for human consumption. Many studies aim to identify the best processing parameters for the preservation of these substances [4,5,6,7,8]. The proximate and mineral composition and the quantification of amino acids, fatty acids, and phytosterols have already been described in the literature [2]. Borges et al. [9] obtained total carotenoids (TC) quantification of around 536 μg/g and 379 μg/g of TC for the Jambu cultivated with organic and mineral fertilization, respectively. However, no studies were found that report the profile of these compounds in that plant.

In leafy vegetables, all-*trans*-lutein is naturally found as the major compound and its ingestion is important to maintain several functions in the body, including visual acuity [10]. According to the Joint FAO/WHO Expert Committee on Food Additives, the acceptable daily intake (ADI) of all-*trans*-lutein is from 0 to 2 mg/kg of body weight [11]. All-*trans-*β-carotene is also one of the main carotenoids present in green leaves. Its daily intake is essential for health, as it is the largest representative of the precursors of vitamin A carotenoids [5]. Thus, these compounds play several important roles in the body, mainly as antioxidant agents [12]. However, sensitivity to high temperatures compromises the recommended daily intake [4].

In this sense, the study of the stability of carotenoids present in Jambu leaves is a parameter that aids the application of these compounds in functional foods since, when incorporated into organic solutions such as edible oils, for example, they will be more bioavailable than carotenoids of a plant cell matrix. However, there are several stability problems that need to be controlled for the carotenoids to be used successfully. When placed in organic solutions, they are less stable than the carotenoids naturally available in vegetable cells [13].

Considering the nutritional importance of carotenoids, this work proposed building drying curves with Jambu leaves (*Acmella oleracea*) at different temperatures and evaluating the effect of heating on the profile and retention of carotenoids. The result of this investigation may provide parameters for obtaining a powdered product based on Jambu, with less impact on the content of carotenoids and with great possibilities for application by the pharmaceutical, cosmetic, and food industries among others.

## 2. Materials and Methods

### 2.1. Chemicals and Reagents

All chromatographic-grade carotenoid analytical standards were purchased from Sigma-Aldrich (St. Louis, MO, USA) and showed a purity degree of at least 95%. The water used in the experiment was purified by reverse osmosis using the Milli-Q system (Millipore Corp., Milford, MA, USA). The other reagents and salts used were of analytical grade, showed at least a 95% purity degree, and were purchased from the following manufacturers: methyl-tert-butyl ether (MTBE) and methanol (MeOH) from Sigma-Aldrich (St. Louis, MO, USA); acetone from Dynamics (São Paulo, Brazil); celite from Synth (São Paulo, Brazil); and petroleum ether, ethyl ether and ethanol from Scientific Êxodo (São Paulo, Brazil).

### 2.2. Jambu Leaves

The collection of Jambu plants was from sixteen different orchards in the municipalities of Ananindeua, Barcarena, Marituba, and Santa Izabel in Pará, Brazil. All producers authorized the study through a Term of Free and Informed Consent (TFIC). The research was registered in the National System for the Management of Genetic Heritage and Associated Traditional Knowledge (SISGEN) under the code A89EDD3. For analyses, a total of approximately 2.88 kg of Jambu leaves were used, with 180 g from each orchard. All plants were collected after 30 days of planting.

The botanical identifications of the species were carried out by the herbarium Prof. Dr. Marlene Freitas da Silva (MFS) of the State University of Pará (UEPA) and deposited as *Acmella oleracea* (L.) R.K. Jansen. The geographic coordinates of the exsiccate collection sites were obtained through the Google Earth Pro software (version 7.1, Google, Mountain View, CA, USA).

### 2.3. Quantification of Total Carotenoids (TC)

The TC content was determined in leaves collected from 16 locations, considered the largest producers of Jambu and also belonging to the producers who agreed to collaborate with the research. For the analysis, *in natura* samples were frozen with liquid nitrogen and freeze-dried under vacuum (T < −60 °C) in a freeze-drier (Alpha 2-4 LDplus, CE Christ, Osterode am Harz, Germany) for 36 h, the time required for leaves to reach 10% moisture. Then, they were ground in an analytical knife mill (A11 basic, IKA^®^, Staufen, Germany), vacuum packed, and stored at −10 °C, protected from light.

The TC content was determined according to Rodriguez-Amaya [14]. The freeze-dried Jambu leaves (0.5 g) were subjected to an exhaustive extraction process by maceration with acetone and celite. The extract obtained was subjected to liquid-liquid partition with petroleum ether/ethyl ether (1:2, *v*/*v*), washed with distilled water, and saponified with KOH (10%) in methanol (1:1, *v*/*v*) for 16 h. Then, the extracts were again partitioned and evaporated in a rotary evaporator (Q344B2, Quims, São Paulo, Brazil) under vacuum (T < 35 °C). The dry extract of carotenoids was solubilized in ethanol up to a volume of 10 mL. The absorbance reading was obtained at 445 nm in a UV-Visible spectrophotometer (EVO60, Thermo Fisher Scientific, Massachusetts, EUA). The TC content was calculated using the specific absorption coefficient of lutein in ethanol ($E_{1\mathrm{cm}}^{1\%}$ = 2550). The results were expressed in µg of TC/g of leaves, considering three independent extraction procedures (*n* = 3).

### 2.4. Convective Drying Process and Mathematical Models

The Jambu leaves with the highest content of total carotenoids were selected for the drying experiments. These had an initial relative moisture of 90.7%. Drying was carried out in an air circulation oven (TE-394/3, Tecnal, São Paulo, Brazil) under different temperature conditions (35, 40, 50 and 60 °C) using 3 g of leaves on each plate (half-thickness of the leaves: 0.0007 m). The air speed of 1.4 m/s^−1^ was measured with a digital anemometer (AD-250, INSTRUTERM, São Paulo, Brazil) and all tests were performed in triplicate.

The moisture ratio (MR), to obtain the curves as a function of time, was calculated using Equation (1).

(1) $$MR=\;\frac{X-X_{e}}{X_{0}-X_{e}}$$

where:

*MR* = sample moisture ratio (dimensionless).

*X* = water content of the product (decimal, dry basis).

*X_e_* = equilibrium water content of the product (decimal, dry basis).

*X*_0_ = initial water content of the product (decimal, dry basis).

The experimental data of the drying curves were adjusted using six mathematical equations (Table 1). The non-linear regression analysis (exponential and polynomial types) of these equations were evaluated using Statistica software (version 7.0, Statsoft Inc., Tulsa, OK, USA).

The predicted and experimental values were used to calculate the goodness of fit for different models. The best fit for the model was selected based on the higher coefficient of determination (R^2^), and the lower value of remaining, mean relative error (*P*), mean estimated error (SE), and chi-square test (χ^2^) at a significance level of 0.05 (*p* ≤ 0.05) according to Equations (2)–(4).

(2) $$P=\frac{100}{n}\sum \frac{\left|Y-\hat{Y}\right|}{Y}$$

(3) $$\mathrm{SE}=\sqrt{\frac{\sum {\left(Y-\hat{Y}\right)}^{2}}{\mathrm{DF}}}$$

(4) $$X^{2}=\frac{\sum {\left(Y-\hat{Y}\right)}^{2}}{\mathrm{DF}}$$

where:

Y = experimental value of MR.

Ŷ = estimated value of MR.

n = number of observations.

DF = degrees of freedom of the model.

### 2.5. Determination of Effective Diffusivity and Activation Energy

The effective moisture diffusivity describes the drying characteristics of foodstuff. The effective diffusivity (D_eff_) of Jambu leaves was calculated from the analytical solution of Fick’s second law (Equation (5)) for plane geometry, considering that the mass transfer occurred on both sides of the material.

(5) $$\mathrm{MR}=\frac{8}{\pi^{2}}\overset{\infty }{\underset{n=0}{\sum }}\frac{1}{{\left(2n+1\right)}^{2}}\exp\left[-\frac{{\left(2n+1\right)}^{2}{\;\pi }^{2}.{\;D}_{\mathrm{eff}}\;}{4\cdot L^{2}}t\right]$$

where:

MR = sample moisture ratio.

D_eff_ = effective diffusivity coefficient (m^2^·s^−1^).

*L*: half-thickness of the leaves (m).

n = number of terms in the equation.

t = time (s).

When the drying process is lengthy, the equation can be simplified to calculate the effective diffusivity coefficient from the previous equation. The diffusion coefficient (D_eef_) for drying can be calculated from the slope obtained by plotting the natural logarithm of MR against the drying time (Equation (6)).

(6) $$\mathrm{Slope}=\frac{\pi^{2}\cdot {\;D}_{\mathrm{eff}}}{4\cdot L^{2}}$$

where:

D_eff_ = effective diffusivity coefficient (m^2^·s^−1^).

*L*: half-thickness of the leaves (m).

The energy of activation (E_a_) was calculated using Arrhenius equation (Equation (7))

(7) $$D_{\mathrm{eff}}=D_{0}\exp\left(\frac{-E_{a}}{{\mathrm{RT}}_{a}}\right)$$

where:

D_0_ = pre-exponential factor (m^2^ s^−1^).

E_a_ = activation energy (J mol^−1^).

R = universal gas constant (8.314 J mol^−1^ K^−1^).

T_a_ = absolute temperature (K).

### 2.6. Determination of Carotenoid Profile by HPLC-DAD

The carotenoid profile was determined in samples dried at different temperatures by high-performance liquid chromatography (HPLC) coupled to a diode array detector (DAD), according to the procedure described by Chisté and Mercadante [21]. HPLC-DAD analysis was performed on an Agilent HPLC (1260 Infinity, Agilent, Santa Clara, CA, USA) equipped with a quaternary pump (G1311C), a Rheodyne injection valve with a 20 µL loop, an oven (G1316A), and a DAD detector (G1328C).

Carotenoid extracts were obtained according to the methodology mentioned above for TC (Item 2.3). At the time of analysis, the dry extracts were solubilized and diluted in methanol/MTBE (70:30, *v/v*) and injected into the chromatographic system. The carotenoids were separated on a C_30_ YMC column (5 μm, 250 mm × 4.6 mm) at 29 °C, using a linear gradient of methanol/MTBE from 95:5 to 70:30 in 30 min as the mobile phase, followed by 50:50 in 20 min under 0.9 mL flow. UV-Visible spectra were recorded between 200 and 600 nm, and chromatograms processed at 450 nm. Carotenoids were tentatively identified according to the following combined information: elution order, retention time (RT), co-chromatography with authentic standards, UV-visible spectra, fine structure data (%III/II), peak intensity *cis* (%A_B_/II), maximum absorption wavelength (λ_max_), and comparison with the literature data.

Quantification was performed using six-point analytical curves and the limits of detection (LOD) and quantification (LOQ) were calculated using the parameters of the analytical curves (standard deviation and slope of the straight line) [22]. For xanthophylls, the lutein standard was used (1.5–46.4 μg/mL; R^2^ = 0.98; LOD = 0.19 μg/mL and LOQ = 0.57 μg/mL). For carotenes, the standard β-carotene was used (0.1–14.7 μg/mL, R^2^ = 0.98, LOD = 0.2 μg/mL and LOQ = 0.6 μg/mL). Vitamin A content was calculated using the NAS-IOM conversion factor, which considers 1 μg of retinol activity equivalent (RAE) corresponding to 12 μg of all-*trans*-β-carotene (a molecule with 100% activity) and 24 μg of 9-*cis*-β-carotene (a molecule with 50% activity) present in the extract [23]. The carotenoid content was expressed in μg/g of sample (dry basis), considering three independent extraction procedures (*n* = 3).

### 2.7. Statistical Analysis

The mean, standard deviation, and analysis of variance (ANOVA) results were expressed at a significance level of 5%. The Tukey test was used to compare the means, and the adjustments of the mathematical models of the drying kinetics to the experimental data were performed using the Statistica software (version 7.0, Statsoft Inc., Tulsa, OK, USA).

## 3. Results and Discussion

### 3.1. Content of Total Carotenoids in Jambu Leaves

The contents of carotenoids quantified in freeze-dried Jambu leaves showed 72% variation (Table 2). The highest quantified value (618 μg/g) did not present a statistical difference from the other nine samples of Jambu analyzed (nº 1, 2, 6, 8, 9, 10, 11, 13 and 16). In addition, the levels observed in these samples were like those reported in the study of Akdaş and Bakkalbaşi [24] for cabbage leaves subjected to different cooking methods (boiling, 556 μg/g; steam cooking, 568 μg/g; microwave, 559 μg/g; and frying, 402 μg/g).

The literature reports that several factors can influence the composition of carotenoids in plants such as temperature, soil conditions, type of cultivar, fertilization, nutrient availability, and even interaction with other plants [5]. In the study of Borges et al. [9], for example, Jambu plants cultivated with different fertilizers showed differences in carotenoid contents. In leaves grown with organic fertilization, the carotenoid content was 498 and 536 μg/g; for mineral fertilization, the values were 379 and 364 μg/g. It is important to emphasize that despite the interference of these conditions, Jambu leaves have high amounts of carotenoids that can provide many possibilities for uses in the pharmaceutical, cosmetic, and food industries among others, and this result highlights the nutritional importance of these leafy vegetables.

### 3.2. Drying Kinetics of Leaves

**Figure 1:**
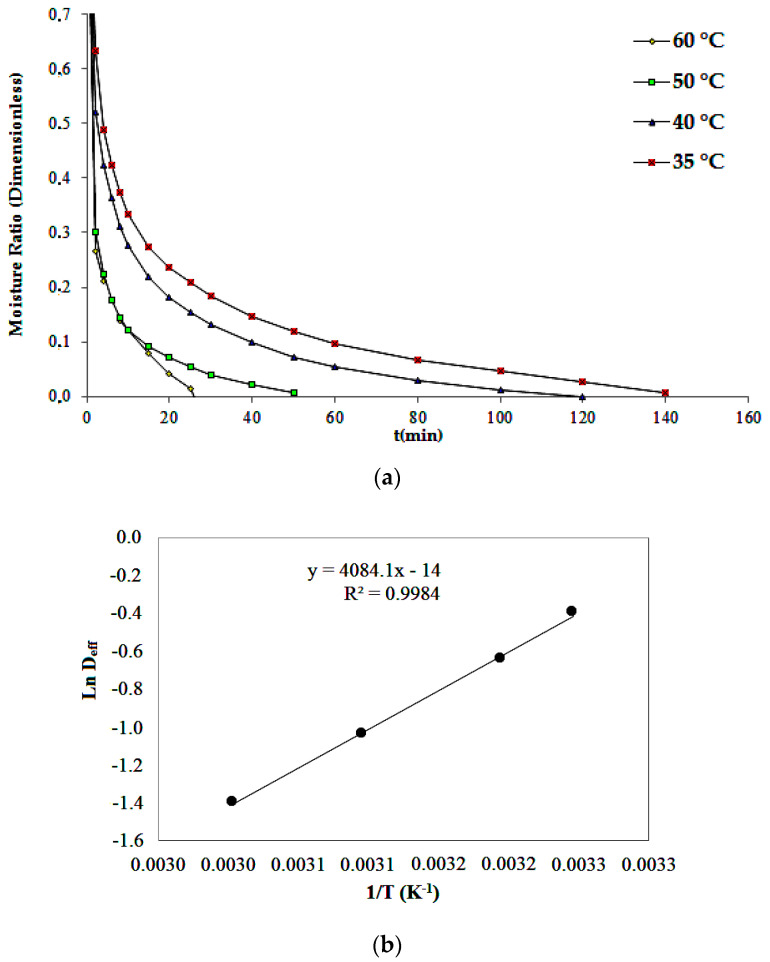
Kinetic drying curves of Jambu leaves at different temperatures (**a**) and natural logarithm of effective diffusivity (D_eff_) as a function of drying air of the inverse of temperature (**b**).

Figure 1 presents the drying curves of Jambu leaves at the four studied temperatures and the effective diffusivity (D_eff_) as a function of the drying air temperature. The drying process was interrupted when the moisture content of the leaves reached 10% (14 h of drying); 9.6% (11 h drying); 8.9% (8 h of drying) and 9.7% (4 h of drying) of the initial value for the temperatures of 35, 40, 50 and 60 °C, respectively. At the end of the process, the dry samples were analyzed for the carotenoid profile by HPLC-DAD.

In comparison with the freeze-drying process (36 h of drying), the temperatures of 35, 40, 50 and 60 °C reduced the drying time by 61, 69, 78 and 89%, respectively. When compared to drying at 35 °C, temperatures of 40, 50 and 60 °C reduced drying time by 21, 43 and 71%, respectively. These results are interesting from an industrial point of view, as they represent cost savings during the processing of products made with Jambu leaves and, consequently, increase the commercial expansion of the product.

Table 3 summarizes the values obtained for each parameter of the kinetic models used to evaluate the drying behavior of Jambu leaves. The results for the coefficient of determination (R^2^), mean relative error (*P*), mean estimated error (SE), and chi square test (χ^2^) demonstrate that only the Page [15] model presents a proper fit to describe the drying process. In a study carried out with *Momordica charantia* L. leaves, the Page model was also the one that best represented the drying kinetics [25]. The drying coefficient *k* increases with increasing temperature, indicating that the Page model is related to the effective diffusivity. The latter presented values ranging from 1.5 × 10^−5^ to 4.0 × 10^−5^ m^2^/s during drying and correspond to temperatures of 35 and 60 °C. The variation in the effective diffusion coefficient occurs due to the rise in temperature, which consequently increases the vibration level of the water molecules and contributes to a faster diffusion [25].

The results of drying Jambu leaves also demonstrate that the activation energy for water diffusion was 34 kJ/mol^−1^, a typical value for agricultural products that normally present values from 13 to 110 kJ/mol^−1^ [26]. This result is significant because E_a_ is a barrier that must be overcome to trigger the diffusion process. In drying processes, the lower the activation energy, the greater the diffusivity of water in the product [25]. In the study of Gomes et al. [27], it was observed that E_a_ obtained during the drying of the crushed Jambu mass corresponds to 17 kJ/mol^−1^, and that the effective diffusion coefficient is influenced by the increase in the thickness of the material and by the rise in temperature.

### 3.3. Carotenoid Profile of Jambu Leaves and the Effect of Drying Temperature

**Figure 2:**
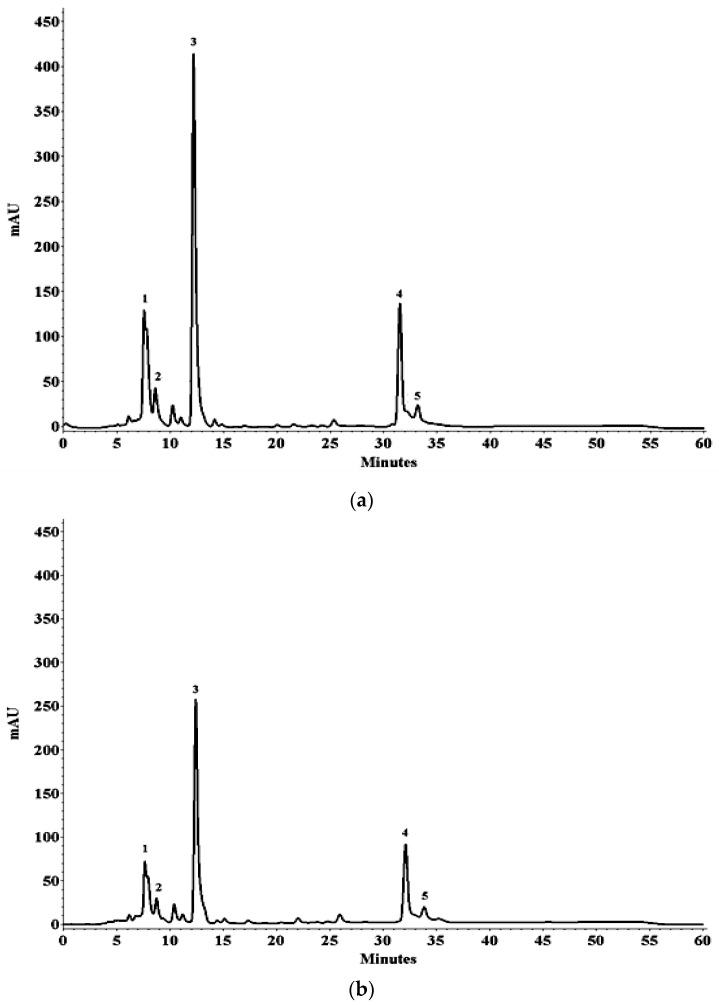
Chromatogram obtained by HPLC-DAD of carotenoid extracts of Jambu leaves dried by freeze-drier (**a**) and in an oven at 35 °C (**b**). Peak characterization (1, 2, 3, 4 and 5) is given in Table 4.

Analysis by HPLC-DAD allowed the identification and quantification of five peaks of carotenoids in dried Jambu leaves (Figure 2, Table 4). Peak 1 was identified as a mixture of *cis*-carotenoid with all-*trans*-violaxanthin, due to its λ_max_, fine structure patterns, and *cis* peak intensity characteristic of these compounds [28]. Peak 2 was identified as all-*trans*-luteoxanthin. These three compounds were also identified in chicory leaves in the study by Leitão et al. [28] and the spectral characteristics are like those obtained in the chromatogram of Jambu leaves. Peaks 3 and 4 were positively identified as all-*trans*-lutein and all-*trans*-β-carotene, respectively, by comparison with authentic standards and fine structure values (77 and 23%), λ_max_ (444 and 450 nm), and retention times in column C_30_ compared to the literature data for green leafy vegetables [12,28]. Peak 5 was identified as 9-*cis*-β-carotene compared to spectral data and literature information [12]. This compound usually has a longer retention time than all-*trans*-β-carotene in the C_30_ column and the *cis* peak intensity is low compared to other possibilities of isomers (13 and 15-*cis*), in addition to absorption and characteristic thin structure [6]. Therefore, the profile found is similar to the results reported in the literature for leafy vegetables [10], as it contains all-*trans*-lutein as the major compound. The total of carotenoids quantified by HPLC-DAD is higher than those found in the literature for chicory leaves (*Eryngium foetidum*, 553 μg/g), pariri (*Arrabidaea chica*, 561 μg/g), and pumpkin pulp (*Cucurbita maxima*, 855 μg/g) [8,12,28].

Regarding the retention of carotenoids, the freeze-drying process preserved a significant content (*p* > 0.05) of carotenoids in Jambu leaves compared to oven drying, with emphasis on the concentration of all-*trans*-lutein (599 µg/g), which was three times higher than the all-*trans*-β-carotene content (196 µg/g). The concentrations of carotenoids in the kiln-dried leaves showed significant differences between the temperatures studied, with drying at 35 °C being the one that most preserved the carotenoids (Table 4). The *cis*-carotenoid mix’s degradation percentages with all-*trans*-violaxanthin (peak 1) ranged from 62 to 96%. The higher temperatures caused the highest degradation rates of this mixture of carotenoids. All-*trans*-luteoxantina also shows high sensitivity to temperature rises, as at 35 °C there was 75.5% degradation (Table 4). The values were not detected at other temperatures, as they were below the LOQ. The percentages of degradation of all-*trans*-lutein were 31, 76, 58 and 54% for the temperatures of 35, 40, 50 and 60 °C (Table 4), respectively, indicating that the higher the temperature, the higher the degradation rate, however at 40 °C this decrease is more intense, suggesting the action of other factors, such as the action of enzymes. According to Zhao-Peng et al. [29], in the range of 40 to 50 °C, higher rates of degradation of carotenoids can occur due to the activation of lipolytic enzymes, which act on nonpolar compounds causing enzymatic degradation. Furthermore, among the identified carotenoids, xanthophylls showed greater sensitivity to temperature increases. This fact may be associated with the chemical structure composed of hydroxyls that allow greater interactions with the evaporation water [4].

The content of carotenoids in vegetables can also influence the degradation rate. In the study by Ouyang et al. [8], for example, the levels of carotenoids and carotenoid esters in pumpkin slices subjected to drying with hot air (60–100 °C, 6–17 h) were evaluated and 76 to 98% of the lutein diesters were retained in the dry end products, indicating a lower degradation rate compared to Jambu. Therefore, even though pumpkin has a four times lower lutein content than Jambu, there was greater preservation of this carotenoid. This indicates that when there are higher concentrations of carotenoids, degradation rates increase.

The degradation percentages for all-*trans*-β-carotene were 4, 56, 35 and 6% at temperatures of 35, 40, 50 and 60 °C (Table 4), respectively, indicating that the temperature range from 40 to 50 °C does not preserve a significant amount of this carotenoid. All-*trans*-*β*-carotene is more preserved in thermal processing at 35 and 60 °C. The same behavior was observed for its 9-*cis*-β-carotene isomer. In general, carotenes were more stable than xanthophylls. This behavior is probably due to the chemical structure of a long polyene chain with conjugated double bonds and no polar substituents. This structure guarantees the hydrophobicity of most carotenoids, providing less interaction with the evaporation water and favoring stability during the drying process [14].

All-*trans*-β-carotene and its 9-*cis*-β-carotene isomer present in Jambu are responsible for the activity of provitamin A by having an unsubstituted β-ionone ring and an attached polyene side chain of at least eleven carbons [14]. In the freeze-dried leaves, 18 μg RAE/g were quantified and the value is similar to that found by Leitão et al. [28] for *Eryngium foetidum* leaves (17 μg RAE/g). However, the drying process in an oven increased the degradation rate from 9.4 to 59%, with the temperature of 35 °C being the one that most assured the retention of these compounds, as the content did not show any statistical difference when compared to the freeze-dried sheets.

According to the work of Britton and Khachik [30], a raw material can be classified as a low (0–1 μg/g), moderate (1–5 μg/g), high (5–20 μg/g), or very high (>20 μg/g) source of carotenoids. In this sense, freeze-dried Jambu leaves and those dried in an oven at 35 °C are classified as very high sources of all-*trans*-lutein, all-*trans*-β-carotene, and all-*trans*-violaxanthin. These results are interesting for the scientific community and industry, as carotenoids have shown preventive effects against several diseases [10]. All-*trans*-lutein, for example, plays a vital role in maintaining the normal visual function of the human macula, and all-*trans*-β-carotene, in addition to its provitamin A activity, has a recognized antioxidant activity capable of attenuating the harmful effects of oxidizing reactive species [10,12]. However, the presence of carotenoids in Jambu leaves provides a potential for the development of bioactive ingredients for functional foods, nutraceuticals, and pharmaceuticals, in addition to contributing to the recommended daily intake of carotenoids in the human diet. The results also highlight the importance of creating strategies to develop the production of dry Jambu and thus encourage the consumption of this vegetable in different regions to ensure that the population has more significant contact with the functional potential of this raw material.

The study of the drying process of Jambu is essential for the extraction of carotenoids. Different plant matrices can exhibit different behaviors during drying and the experimental curves produce results that provide a more accurate evaluation of the process. The curves provide basic and fundamental information for determining the maximum residual moisture that a food or natural product must contain for safe storage, avoiding microbiological activity, and enzymatic oxidation processes. Studying the drying process of Jambu associated with the stability of carotenoids contributes to the development of more effective technologies to obtain these compounds, in addition to boosting different applications for the food, cosmetics, and pharmaceutical industries.

## 4. Conclusions

The content of carotenoids found in Jambu leaves classifies the vegetable as a very high source of carotenoids. The Page model well represented the leaf drying kinetics, and the results demonstrate that the activation energy for water diffusion is typical of agricultural products (34 kJ/mol^−1^), with a reduction in drying time that represents more significant cost savings and greater market competitiveness for products made with Jambu leaves.

The carotenoid profile resembles those found for leafy vegetables with all-*trans*-lutein as the major compound. Variations in drying temperature did not interfere with the chromatographic profile of carotenoids, but they caused significant decreases in the retention of these compounds and, consequently, in the content of vitamin A. However, the convective drying of Jambu leaves at 35 °C ensured less degradation of the carotenoids. The dry final product presented a significant amount of all-*trans*-lutein to meet the daily requirements recommended by the FAO/WHO (0 to 2 mg/kg of body weight) for the human diet and has a considerable vitamin A content.

## Figures and Tables

**Table 1:** Mathematical equations used for drying curves.

Equations		Author
Page	$MR=exp\;\left(-k\;t^{n}\right)$	[15]
Henderson and Pabis	$MR=a\exp\;\left(-k\;t\right)$	[16]
Newton	$MR=exp\;\left(-k\;t\right)$	[17]
Logarithmic	$MR=a\exp\;\left(-k\;t\right)+c$	[18]
Thompson	$MR=\exp\;\left\{\frac{\left[-a-{\left(a^{2}+4\;b\;t\right)}^{0.5}\right]\;}{2\;b}\right\}$	[19]
Diffusion approach	$MR=a\exp\left(-k\;t\right)+\left(1-a\right)\exp\left(-k\;b\;t\right)$	[20]

*a*, *b*, *c*, *n*: model coefficients; *k*: drying constant (s^−1^); MR: sample moisture ratio; *t*: drying time (s).

**Table 2:** Content of total carotenoids, geographic coordinates of collection sites, and record number of exsiccates from 16 Jambu plants obtained from different producers.

N°	Total Carotenoids (μg/g) ^A^	Producer/Municipality	Geographical Coordinates	Exsiccate Registration ^B^
1	391 ± 11 ^a,b,c,d,e,f^	J.O.C./Ananindeua	(1°19′20.34″ South) (48°23′25.25″ West)	MFS008250
2	398 ± 75 ^a,b,c,d,e,f^	G.M.P./Ananindeua	(1°19′21.52″ South) (48°23′29.94″ West)	MFS008251
3	237 ± 41 ^e,f^	R.T./Ananindeua	(1°19′39.68″ South) (48°23′14.65″ West)	MFS008252
4	346 ± 8 ^b,c,d,e,f^	C.C.A./Ananindeua	(1°19′38.55″ South) (48°23′07.83″ West)	MFS008253
5	175 ± 16 ^f^	L.S.P./Ananindeua	(1°19′37.62″ South) (48°23′07.94″ West)	MFS008261
6	488 ± 65 ^a,b,c,d^	J.O.G.O./Ananindeua	(1°19′37.70″ South) (48°23′07.40″ West)	MFS008262
7	409 ± 69 ^b,c,d,e,f^	M.N.C.S./Santa Izabel	(1°16′37.07″ South) (48°05′51.48″ West)	MFS008337
8	472 ± 37 ^a,b,c,d,e^	L. A.B. J./Ananindeua	(1°19′08.75″ South) (48°23′35.63″ West)	MFS008344
9	557 ± 6 ^a,b^	J.M./Ananindeua	(1°19′22.87″ South) (48°23′25.92″ West)	MFS008343
10	576 ± 50 ^a,b^	J. B. N./Santa Izabel	(1°16′26.22″ South) (48°05′23.70″ West)	MFS008335
11	492 ± 177 ^a,b,c,d^	R. B. N./Santa Izabel	(1°16′29.28″ South) (48°05′22.51″ West)	MFS008336
12	313 ± 14 ^c,d,e,f^	M. N. S. N./Santa Izabel	(1°16′29.12″ South) (48°05′27.20″ West)	MFS008339
13	507 ± 26 ^a,b,c^	R.D.C./Santa Izabel	(1°15′40.76″ South) (48°07′15.14″ West)	MFS008448
14	257 ± 13 ^d,e,f^	J.R.M./Barcarena	(1°34′50.88″ South) (48°36′25.08″ West)	MFS008445
15	618 ± 46 ^a^	M.J.C./Marituba	(1°23′12.75″ South) (48°21′03.09″ West)	MFS008447
16	442 ± 77 ^a,c,d,e^	V.E./Ananindeua	(1°23′07.41″ South) (48°21′49.83″ West)	MFS008342
CV (%)	0.3			

^A^ Values with different letters in the same column show a significant difference (*p* ≤ 0.05) and the means were obtained from three independent extraction procedures (*n* = 3); ^B^ All exsiccates were performed in duplicates. Lowercase letters (a, b, c, d, e, f) represent statistical differences between column values.

**Table 3:** Jambu drying adjustment parameters for different mathematical equations at temperatures of 35, 40, 50 and 60 °C.

Equations	T (°C)	Coefficients						Statistical Analysis		
		a	k	n	b	c	R^2^	*P* (%)	SE ×10^−3^	χ^2^ ×10^−3^
Page	35		0.3655	0.4608			0.99	5.91	0.503	0.0003
	40		0.4691	0.4375			0.99	5.26	0.112	0.00001
	50		0.6963	0.4691			0.99	6.52	1.709	0.003
	60		0.9974	0.3616			0.99	8.92	0.600	0.0004
Henderson and Pabis	35	0.8011	0.0734				0.94	47.58	31.767	1
	40	0.8242	0.1127				0.93	50.60	30.900	1
	50	0.9554	0.3861				0.96	68.68	20.130	0.4
	60	0.9695	0.4605				0.96	41.91	16.086	0.3
Newton	35		0.1122				0.91	57.30	43.721	1.9
	40		0.1571				0.92	58.40	39.473	1.6
	50		0.4091				0.96	69.79	20.686	0.4
	60		0.4779				0.96	67.31	18.942	0.4
Logarithmic	35	0.8045	0.1359			0.1029	0.97	43.62	14.815	0.2
	40	0.8154	0.1947			0.0993	0.97	40.57	14.238	0.2
	50	0.9120	0.5675			0.0757	0.98	54.78	6.980	0.05
	60	0.9060	0.7148			0.891	0.98	32.59	4.506	0.02
Thompson	35	0.0023			0.4776		0.99	8.71	1.086	0.001
	40	0.0223			0.5456		0.99	9.42	1.204	0.001
	50	0.0079			0.8051		0.99	25.21	2.077	0.004
	60	0.0054			0.8614		0.99	22.83	2.557	0.007
Diffusion approach	35	0.5844	0.4135		0.0620		0.99	7.65	0.472	0.0002
	40	0.5739	0.7009		0.0560		0.99	42.66	7.347	0.05
	50	0.7338	1.1529		0.0600		0.99	6.40	0.150	0.00002
	60	1.0078	0.4601		0.0233		0.96	44.05	17.731	0.3

T: temperature; a, k, n, b, c: model parameters; R^2^: determination coefficient; *P*: mean relative error; SE: estimated mean error; χ^2^: chi-square test.

**Table 4:** Chromatographic characteristics, UV-Vis, and concentration of carotenoids obtained from leaves from *Acmella oleracea* by HPLC-DAD.

Peak	Carotenoids ^A^	t_R_ (min) ^D^	λ_máx_ (nm) ^E^	%III/II ^F^	%A_B_/II	Freeze-Drying Leaves (µg/g de Leaves) ^C^	Treatment (µg/g Leaves) ^C^			
							35 °C	40 °C	50 °C	60 °C
1	Mix de *cis*-carotenoids + All-*trans*-Violaxanthin ^B^	7.3	328 415 436 462	78	33	271 ± 8 ^a^	102 ± 4 ^b^	18 ± 1 ^c^	19 ± 0 ^c^	11 ± 0 ^c^
		8.1	266 412 436 464	80	0					
2	All-*trans*-Luteoxanthin ^B^	8.6	310 398 420 446	100	0	45 ± 2 ^a^	11 ± 1 ^b^	<LOQ	<LOQ	<LOQ
3	All-*trans*-Lutein	12.2	266 420 444 472	77	0	599 ± 70 ^a^	416 ± 18 ^b^	141 ± 0.2 ^d^	254 ± 9 ^c^	274 ± 9 ^c^
4	All-*trans*-β-carotene ^B^	31.5	276 423 450 476	23	0	196 ± 1 ^a^	188 ± 10 ^a^	86 ± 2 ^c^	127 ± 3 ^b^	184 ± 4 ^a^
5	9-*cis*-β-carotene ^B^	33.2	276 420 448 470	nc ^H^	nc ^H^	34 ± 4 ^a^	30 ± 2 ^a^	3 ± 0 ^d^	11 ± 0 ^c^	17 ± 0 ^b^
Total carotenoids (µg/g leaves)						1145 ± 71 ^a^	748 ± 27 ^b^	247 ± 3 ^e^	411 ± 8 ^d^	486 ± 7 ^c^
A vitamin value (μg RAE/g leaves) ^G^						18 ± 0 ^a^	17 ± 1 ^ab^	7 ± 0 ^d^	11 ± 0 ^c^	16 ± 0 ^b^

^A^ Identification based on UV-visible, C_30_ column retention times, maximum lick (λ_max_), fine structure data, *cis* peak intensity, and data from the literature publications. LOQ: limit of quantification. ^B^ Peaks quantified as All-*trans*-β-carotene equivalents. ^C^ Values with different letters on the same line show a significant difference. ^D^ Column retention time C_30_; ^E^ Maximum absorption peak in the spectrum UV-Vis; ^F^ %III/II: fine spectral structure, defined as the ratio of the height of the absorption peak of the longest wavelength in the spectrum. UV-Vis (designated III) and the mean absorption peak (designated II) taking the minimum between the two peaks as the baseline, multiplied by 100. ^G^ RAE: Retinol activity equivalent. ^H^ nc: incalculable value. Lowercase letters (a, b, c, d, e) represent statistical differences between line values.

## Data Availability

The authors declare that the dataset used and analyzed during the current study can be made available upon reasonable request.
